# The Role of PPARs in Cancer

**DOI:** 10.1155/2008/102737

**Published:** 2008-06-18

**Authors:** Keisuke Tachibana, Daisuke Yamasaki, Kenji Ishimoto, Takefumi Doi

**Affiliations:** ^1^Graduate School of Pharmaceutical Sciences, Osaka University, 1-6 Yamadaoka, Suita, Osaka 565-0871, Japan; ^2^The Center for Advanced Medical Engineering and Informatics, Osaka University, 2-2 Yamadaoka, Suita, Osaka 565-0871, Japan; ^3^Graduate School of Medicine, Osaka University, 2-2 Yamadaoka, Suita, Osaka 565-0871, Japan

## Abstract

Peroxisome proliferator-activated receptors (PPARs) are ligand-activated transcription factors that belong to the nuclear hormone receptor superfamily. PPAR*α* is mainly expressed in the liver, where it activates fatty acid catabolism. PPAR*α* activators have been used to treat dyslipidemia, causing a reduction in plasma triglyceride and elevation of high-density lipoprotein cholesterol. PPAR*δ* is expressed ubiquitously and is implicated in fatty acid oxidation and keratinocyte differentiation. PPAR*δ* activators have been proposed for the treatment of metabolic disease. PPAR*γ*2 is expressed exclusively in adipose tissue and plays a pivotal role in adipocyte differentiation. PPAR*γ* is involved in glucose metabolism through the improvement of insulin sensitivity and represents a potential therapeutic target of type 2 diabetes. Thus PPARs are molecular targets for the development of drugs treating metabolic syndrome. However, PPARs also play a role in the regulation of cancer cell growth. Here, we review the function of PPARs in tumor growth.

## 1. INTRODUCTION

**Figure 1:**
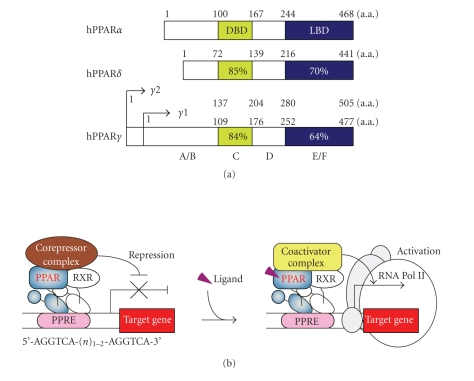
The general features of human PPARs. (a) Structure and functional domain of human PPARs. A/B, C, D, and E/F indicate N-terminal A/B domain containing a ligand-independent activation function 1, DNA-binding domain (DBD), hinge region, and C-terminal ligand-binding domain (LBD), respectively. The number inside each domain corresponds to the percentage of amino acid sequence identity of human PPAR*δ* and PPAR*γ* relative to PPAR*α*. (b) PPAR/RXR heterodimers bind to a PPRE located in the promoter of target genes through the DBD. Unliganded PPAR associates with the corepressor complex. In the presence of ligand, the ligand-bound LBD associates with the coactivator complex.

Peroxisome proliferator-activated receptors (PPARs) are ligand-activated transcription factors that belong to the nuclear hormone receptor superfamily [1]. PPARs bind to a direct repeat of two hexanucleotides, spaced by one or two nucleotides (the DR1 or DR2 motif) as heterodimers with the retinoid X receptor (RXR), and activate several target genes [2–4]. These peroxisome proliferator responsive elements (PPREs) are found in various genes that are involved in lipid metabolism and energy homeostasis,including lipid storage or catabolism, and fatty acid transport, uptake, and intracellular binding [5]. Three subtypes, PPAR*α*, PPAR*δ* (also known as PPAR*β*), and PPAR*γ*, have been identified and these subtypes with a high degree of sequence conservation of each subtype across various species have been characterized. The DNA-binding domains of the three subtypes are 80% identical, while their ligand-binding domains exhibit a lower degree (approximately 65%) of identity (Figure 1). Consistent with this relatively high divergence among the subtype-specific ligand-binding domains, differential activation of PPARs by endogenous and exogenous compounds may account for the specific biological activity of the three PPAR subtypes [6, 7].

PPAR*α* is expressed in the liver, kidney, small intestine, heart, and muscle, where it activates fatty acid catabolism and is involved in the control of lipoprotein assembly [8]. PPAR*α* is activated by several molecules, such as long chain unsaturated fatty acids, eicosanoids, and hypolipidemic drugs (e.g., fenofibrate) [9–12]. PPAR*α* activators have been used to treat dyslipidemia, causing a reduction in plasma triglyceride and elevation of high-density lipoprotein (HDL) cholesterol [13, 14]. PPAR*δ* is expressed ubiquitously and is implicated in fatty acid oxidation, keratinocyte differentiation, wound healing, and the response of macrophages for very low-density lipoprotein [15–19]. PPAR*δ* activators have been proposed for the treatment of metabolic disease and are under clinical trial [20, 21]. There are two PPAR*γ* isoforms: PPAR*γ*1 and *γ*2 [22, 23]. PPAR*γ*2, which is generated by alternative splicing, contains an additional 28 amino acids at the N-terminal compared to PPAR*γ*1. PPAR*γ*3 is a splicing variant of PPAR*γ*1 and gives rise to the same protein [24]. PPAR*γ*2 is expressed exclusively in adipose tissue and plays a pivotal role in adipocyte differentiation, lipid storage in the white adipose tissue, and energy dissipation in the brown adipose tissue [22, 25]. On the other hand, PPAR*γ*1 is expressed in the colon, the immune system (e.g., monocytes and macrophages), and others. Except for the function of PPAR*γ*2 in adipose tissue, PPAR*γ* also participates in inflammation, cell cycle regulation, and other functions [26]. PPAR*γ* is involved in glucose metabolism through the improvement of insulin sensitivity and represents a potential therapeutic target of type 2 diabetes [26]. Indeed, insulin-sensitizing thiazolidinedione (TZD) drugs are PPAR*γ* ligands [27]. Thus PPARs are molecular targets for the development of drugs to treat type 2 diabetes and metabolic syndrome. On the other hand, PPARs also play a role in the regulation of cancer cell growth.

## 2. PPAR*α* AND CANCER

Fibrates, which are relatively weak PPAR*α* ligands, are useful for the treatment of dyslipidemia [7, 9–11]. Fibrates lower serum triglyceride levels and increase HDL levels through the activation of PPAR*α* [5]. PPAR*α* induces lipoprotein lipase (LPL) expression, reduces the expression levels of apolipoprotein C-III (ApoC-III), a natural LPL inhibitor, and stimulates the uptake of cellular fatty acids and the conversion of fatty acids to acyl-CoA derivatives [5, 28, 29]. These catabolism functions are mediated by upregulating the expression of a series of genes-related carbohydrate and lipid metabolism [5, 30]. In addition, PPAR*α* increases the expressions of ApoA-I and ApoA-II, resulting in raising HDL cholesterol levels in humans [31, 32]. Thus PPAR*α* plays a central role in the control of fatty acid and lipoprotein metabolism, and improves plasma lipid profiles. Although peroxisome proliferators have carcinogenic consequences in the liver of rodents, epidemiological studies suggest that similar effects are unlikely to occur in humans [10, 33–36].

Several mechanisms have been proposed to explain the carcinogenesis of peroxisome proliferators in rodents. Peters et al. reported that wild-type mice treated with the Wy-14,643 showed increase of replicative DNA synthesis in hepatic cells and developing liver tumors with 100% incidence, whereas PPAR*α*-null mice were refractory to this effect [37]. Peroxisome proliferators increase the peroxisome volume and number and result in an increase in hydrogen peroxide (H_2_O_2_) levels [38–40]. These effects may be mediated in part by the increased expression of peroxisomal enzymes that produce H_2_O_2_, such as acyl CoA oxidase (ACO) [39–41]. PPAR*α* upregulates the expression levels of ACO via PPRE in the promoter region [42, 43]. A stably transfected African green monkey kidney cells (CV-1) overexpressing rat ACO increased H_2_O_2_ production, formed transformed foci, and grew efficiently in soft agar when the cells were treated with linoleic acid [44]. Furthermore, when these cells were transplanted into nude mice, these cells formed solid tumors [44]. An increase of intracellular levels of H_2_O_2_ could lead to DNA damage via a variety of mechanisms [45]. Any reduced iron present can catalyze the cleavage of H_2_O_2_, via the Fenton reaction, to produce hydroxyl radicals (HO•) [46]. The HO• attacks guanine residues, producing residues of 8-oxo-7,8-dihydroguanine (8-oxoguanine). When DNA synthesis occurs before the 8-oxoguanine is repaired, this damaged base will have a chance to pair with adenine nucleotide, resulting in a mutation in the daughter cells [47]. In addition, antioxidants inhibit ciprofibrate-induced hepatic tumorigenesis by scavenging active oxygen [48]. Thus oxidative stress by peroxisome proliferators acts as a driving force to malignancy. The activation of PPAR*α* also leads to increased hepatocellular proliferation and inhibition of apoptosis. Chronic administration of nafenopin, PPAR*α* agonist, to mice causes significant increase in the liver weight, hepatic DNA synthesis, and the development of hepatocellular carcinomas [49]. Nafenopin treatment of primary cultures of adult rat hepatocytes also stimulated DNA synthesis [50]. Indeed, Peters et al. showed that mRNAs encoding cyclin-dependent kinase (CDK) 1, CDK4, cyclin D1, and c-*myc* and their proteins, which induce cell proliferation, increased in wild-type mice fed by Wy-14,643 but not in PPAR*α*-null mice [51]. Increase of the average liver weight and the levels of mRNAs encoding cell cycle regulation, such as CDK4, proliferating cell nuclear antigen (PCNA) and cyclin B1, were also found in wild-type mice fed by bezafibrate, the less specific PPAR*α* agonist, and these effects were not found in PPAR*α*-null mice [52]. Moreover, the treatment of the primary culture of rat hepatocytes and the rat hepatoma cell line, FaO, with nafenopin suppressed apoptosis [53, 54]. Thus the activation of PPAR*α* leads to the increase of oxidative stress, induction of cell proliferation and inhibition of apoptosis, indicating that PPAR*α* increases hepatocarcinogenesis in mice.

A number of experimental observations suggest that there is a species difference between rodents and humans in the response to PPAR*α* agonists, although the functional differences of PPAR*α* derived from species are not clear (Table 1). One possible explanation for the difference is the expression levels of PPAR*α* in the liver. The expression levels of PPAR*α* in human liver are approximately one order less than that observed in mouse liver [55]. Small expression levels of PPAR*α* could allow PPREs to be occupied by other members of the nuclear receptor superfamily,such as RXR, the chicken ovalbumin upstream promoter transcription factor I (COUP-TFI), COUP-TFII, hepatocyte nuclear factor-4 (HNF4), retinoic acid receptor (RAR), and thyroid hormone receptor (TR), and affect responsiveness to peroxisome proliferators [56–62]. We and others have shown that elevated expression of PPAR*α* in HepG2 cells dramatically increased the expression of several target genes, such as 3-hydroxy-3-methylglutaryl-CoA synthase 2 (mitochondrial) (HMGCS2), carnitine palmitoyltransferase 1A (CPT1A), and long chain fatty acyl-CoA synthetase (ACS) [30, 63, 64]. In this way, the lower expression levels of PPAR*α* in human liver might contribute to holding down peroxisome proliferation and subsequent pathologic effects. Another explanation is that several PPAR*α* variants, which lack the entire exon 6 or contain mutations, are detected in human cells and these variants act as a dominant negative regulator of PPAR-mediated gene transcription [55, 65, 66]. But this has not been found in rodents yet. One PPAR*α* variant containing the mutation prevents the suppression of hepatocyte apoptosis by nafenopin [55, 65, 66]. Thus the expression levels of PPAR*α* variants might affect the response to peroxisome proliferators. Next, there appears to be sequence differences in the PPRE found in the promoter region of ACO. Osumi et al. identified ACO to be a direct PPAR*α* target gene and a functional PPRE located in the proximal promoter of the rat ACO gene [42]. In contrast to the rodent ACO gene promoter, the human ACO gene promoter differs at three bases within the PPRE from the rat ACO promoter and appears refractory to PPAR*α* [42, 67, 68]. This human PPRE was unable to drive peroxisome proliferators-induced gene transcription in cell-based assays [67–69]. Indeed, human liver cell lines and primary hepatocytes did not induce ACO mRNA by treatment with fibrates or other PPAR*α* agonists [63, 64]. A similar pattern, such differences between human and other species, was observed in the expression of ApoA-I gene [31]. Fibrates influence the ApoA-I gene expression, raising it in humans, and lowering it in rodents. These differences are due to a combination of two distinct mechanisms implicating the nuclear receptors PPAR*α* and Rev-erb*α*, a negative regulator of gene transcription [31]. The species-distinct regulation is due to sequence differences in *cis*-acting elements in their respective promoters leading to repression by Rev-erb*α* of rat ApoA-I and activation by PPAR*α* of human ApoA-I. There is a positive PPRE in the human ApoA-I promoter but not in rats. The expression of Rev-erb*α* is induced by fibrates [3, 31]. In the case of rat, this induction leads to the repression of the ApoA-I gene expression through an Rev-erb*α* response element. On the other hand, there is no Rev-erb*α* response element in the human ApoA-I gene [31]. Thus the sequence differences in *cis*-acting elements cause the species-distinct regulation of target genes expression by peroxisome proliferators. However, the mechanism of the species differences is not known in detail.

To determine the mechanism of species difference in response to peroxisome proliferators, Gonzalez et al. generated a liver-specific PPAR*α* humanized mouse line (hPPAR*α* ^TetOff^ mice) in which the human PPAR*α* was expressed in the liver in a PPAR*α*-null background under the control of the tetracycline (Tet) responsive regulatory system [70–72]. The expression of several target genes encoding peroxisomal and mitochondrial fatty acid metabolizing enzymes were elevated in hPPAR*α* ^TetOff^ mice fed Wy-14,643 or fenofibrate, resulting in the decrease of serum triglycerides [70, 73]. However, the expressions of various genes involved in cell cycle regulation (PCNA, c-*myc*, CDK1, CDK4, and cyclins) in the liver were unaffected by Wy-14,643. In addition, hPPAR*α* ^TetOff^ mice were resistant to Wy-14,643-induced hepatocarcinogenesis [70, 73]. Recently, Shah et al. showed that Wy-14,643 regulated mice hepatic MicroRNA (miRNA) expression via a PPAR*α*-dependent pathway [74]. miRNAs are a class of nonprotein-coding, endogenous,small RNAs, and regulate gene expression by translational repression and mRNA cleavage [75]. Some miRNAs regulate cell proliferation and apoptosis processes that are important in cancer formation [76]. The activation of PPAR*α* with Wy-14,643 inhibits the expression of miRNA let-7C, which functions as a tumor suppressor gene [74]. let-7C degrades c-*myc* mRNA by binding to 3' untranslated region (UTR) of the c-*myc* gene. Treatment of mice with Wy-14,643 showed that let-7C expression was decreased and a subsequent increase in c-*myc* was observed. Following an increase in c-*myc*, the levels of the oncogenic mir-17 miRNA cluster were increased [74]. In this way, inhibition of the let-7C signaling cascade may lead to increased hepatocellular proliferation and tumorigenesis. In contrast, hPPAR*α* ^TetOff^ mice do not exhibit downregulation of let-7C and induced c-*myc* and mir-17 expression [74]. Furthermore, Yang et al. generated another type of PPAR*α* humanized mice, hPPAR*α* ^PAC^ mice, that has the complete human PPAR*α* gene sequence including 5' and 3' flanking sequences on a P1 phage artificial chromosome (PAC) genomic clone, introduced onto the mouse PPAR*α*-null background [71]. Upon treatment with the peroxisome proliferators (Wy-14,643 or fenofibrate), hPPAR*α* ^PAC^ mice exhibited peroxisome proliferation, lowering of serum triglycerides, and induction of PPAR*α* target genes encoding enzymes involved in fatty acid metabolism. However, let-7C expression was not decreased and the expression levels of c-*myc*, cyclin D1 and CDK4 were not increased [71]. Thus these observations suggest that the species differences in response to peroxisome proliferators could be due in part to a differential ability of the mouse and human PPAR*α* to suppress let-7C gene expression [74]. However, the mechanism involved in PPAR*α*-dependent repression of let-7C is unclear. The differences between the wild-type mice and PPAR*α* humanized mice could be caused by the structural differences between human and mouse PPAR*α* and differential coactivator recruitment. However, additional investigation is required to better understand and clarify the mechanism of action of PPAR*α* in causing hepatocarcinogenesis.

## 3. PPAR*δ* AND CANCER

The role of PPAR*δ* in oncogenesis is controversial, especially in colon cancer. Some reports show that PPAR*δ* promotes tumorigenesis by increasing cell proliferation. Indeed, the levels of PPAR*δ* mRNA are increased in both human and rodent colorectal carcinomas [77, 78]. PPAR*δ* is a potential downstream target gene of the adenomatous polyposis coli (APC)/*β*-catenin/T cell factor-4 (TCF-4) pathway [77]. APC is a tumor suppressor gene and is mutated in familial adenomatous polyposis (FAP) and most sporadic colorectal tumors [79–83]. *β*-catenin, which binds to APC and axin in a large protein complex, can be phosphorylated by glycogen synthase kinase-3*β* (GSK3*β*) and is followed by ubiquitination and degradation. Mutation of APC results in the accumulation of *β*-catenin, which in turn translocates to the nucleus and associates with the transcription factor TCF-4 [84]. The *β*-catenin-TCF-4 transcription complex increases the transcription of growth-promoting genes, such as c-*myc* and cyclin D1 [85, 86]. The *β*-catenin-TCF-4 transcription complex also activates the human PPAR*δ* promoter activity via TCF-4 binding sites, namely, APC suppresses the PPAR*δ* expression through the degradation of *β*-catenin [77]. K-Ras mutation is found in colorectal cancer [80, 87]. Activation mutations in Ras result in the activation of the mitogen-activated protein kinase (MAPK) pathway and induce tumor growth and progression [88]. The expression levels and activity of PPAR*δ* were increased by the induction of mutated K-Ras in conditionally K-Ras-transformed rat intestinal epithelial cells [89]. Thus PPAR*δ* is also a downstream target gene of Ras/Raf/MAPK and extracellular signal-regulated kinase (ERK) kinase (MEK)/ERK pathway [89]. In this way, PPAR*δ* may play a role in colon cancer.

Epidemiological studies have shown that nonsteroidal anti-inflammatory drugs (NSAIDs), such as aspirin, indomethacin, and sulindac, reduce the overall number and size of adenomas in patients with FAP. Healthy individuals using NSAIDs regularly can lead to a 40–50% reduction in the relative risk of developing colon cancer [90]. NSAIDs inhibit cyclooxygenase (COX) activity and thereby reduce prostaglandin synthesis [91]. COX is a key enzyme in arachidonic acid metabolism and prostaglandin production. COX catalyzes a two-step reaction that converts arachidonic acid to prostaglandin H_2_ (PGH_2_), which in turn serves as the precursor for the synthesis of all biologically active prostaglandins, including PGD_2_, PGE_2_, PGF_2_ *α*, prostacyclin (PGI_2_), and thromboxane A_2_ (TXA_2_) [92]. COX exists in two isoforms that are encoded by two separate genes. COX-1 is constitutively expressed in most tissues, on the other hand, the expression of COX-2 is normally low or absent in most tissues but is rapidly upregulated by proinflammatory cytokines [93]. Expression of COX-2 is also elevated in colorectal cancer and in a subset of adenomas [94]. Moreover, since both the introduction of the knockout mutation of the COX-2 gene into *A* *p* *c* ^Δ716^ mice, a model of human FAP, and treating *A* *p* *c* ^Δ716^ mice with NSAIDs reduce the development of intestinal tumors, COX-2 inhibitors have been considered as therapeutic agents for colorectal polyposis and cancer [95]. He et al. reported that NSAIDs inhibited the transcriptional activity of PPAR*δ* by disruption of the DNA binding ability of PPAR*δ*/RXR heterodimers, and ectopic expression of PPAR*δ* in the human colorectal cancer cell line, HCT116, protected the cells from sulindac-induced apoptosis [77]. PPAR*δ* and COX-2 mRNA are expressed in similar regions in human colon cancer, and the stable PGI_2_ analog, carbaprostacyclin (cPGI), acts as a PPAR*δ* ligand [11, 78]. Indeed, ectopic expression of COX-2 and PGI synthase (PGIS) in the human osteosarcoma cell line, U2OS, produced high levels of endogenous PGI_2_ and transactivation of PPAR*δ* [78]. PGE_2_ levels are also elevated in human colorectal cancers and adenomas, and PGE_2_ increases the growth and motility of colorectal carcinoma cells [96, 97]. D. Wang et al. showed that PGE_2_ promoted resistance to serum starvation-induced apoptosis of cultured human colon carcinoma cells, LS-174T, through indirectly upregulation PPAR*δ* transcriptional activity via a phosphotidylinositol-3-kinase (PI3K)-Akt pathway [98]. Furthermore, PGE_2_ accelerates intestinal adenoma growth of *A* *p* *c* ^min^ mice, a model of human FAP that harbors a mutation in the *apc* gene, via PPAR*δ* [98]. Xu et al. showed that PGE_2_ activated cytosolic phospholipase A_2_ *α* (cPLA_2_ *α*) through PI3K or MAPK pathway, and subsequently cPLA_2_ *α* enhanced PPAR*δ* activity in the human cholangiocarcinoma cells [99]. They also showed that PPAR*δ* enhanced COX2 expression and PGE_2_ production. This positive feedback loop may play an important role in cholangiocarcinoma cell growth, although it is not known whether this kind of positive feedback loop exists in the colorectal cancer cells [99]. Thus PPAR*δ* induces the cell proliferation through the inhibition of apoptosis. However, sulindac sulfide induces apoptosis not only in wild-type HCT116, but also in HCT116 PPAR*δ*-null cell lines [100]. On the basis of these observations, although NSAIDs may reduce tumorigenesis through the inhibition of PPAR*δ* activity, PPAR*δ* is not a major mediator of sulindac-mediated apoptosis.

Recent evidence supports the hypothesis that PPAR*δ* promotes tumor progression. HCT116 PPAR*δ*-null cell lines grew slightly more slowly than wild-type HCT116 cells, and exhibited a decreased ability to form tumors compared with wild-type mice when inoculated as xenografts in nude mice [100]. Gupta et al. showed that exposure of *A* *p* *c* ^min^ mice to 10 mg/kg of GW501516, a high-affinity PPAR*δ*-selective agonist, led to a two-fold increase in polyp number in the small intestine [101]. The most prominent effect was on polyp size, mice treated with the PPAR*δ* activator had a five-fold increase in the number of polyps larger than 2 mm, suggesting that PPAR*δ* activation primarily affected the rate of polyp growth rather than initiating polyp formation. Pretreatment of wild-type HCT116 cells with GW501516 significantly suppressed serum starvation-induced apoptosis in a dose-dependent manner, but not HCT116 PPAR*δ*-null cells [101]. Furthermore, D. Wang et al. showed that *PPARd* ^−/−^/*A* *p* *c* ^min^ mice decreased intestinal adenoma growth and inhibited the tumor-promoting effect of GW501516 [102]. They also showed that PPAR*δ* activation with GW501516 upregulated vascular endothelial growth factor (VEGF) transcription, expression, and peptide release in intestinal epithelial tumor cells, and subsequently activated PI3K-Akt signaling [102]. Similar results were obtained in the human endothelial cells [103, 104]. Piqueras et al. showed that GW501516 induced VEGF mRNA and peptide release, and thus PPAR*δ* induced endothelial cell proliferation and angiogenesis [103]. Stephen et al. showed that the activation of PPAR*δ* resulted in increased expression of VEGF and its receptor fms-related tyrosine kinase 1 (FLT-1), and they suggested that PPAR*δ* might initiate an autocrine loop for cellular proliferation and possibly angiogenesis [104]. These results demonstrate that VEGF mediates the antiapoptotic effects of PPAR*δ* in intestinal epithelial tumor cells by activating the PI3K-Akt cell survival pathway, and the VEGF autocrine loop plays an important role in cell survival. Diminished apoptosis is also linked to downregulated 15-lipoxygenase-1 (15-LOX-1) expression in colorectal cancer cells. 13-S-hydroxyoctadecadienoic acid (13-S-HODE), which is the primary product of 15-LOX-1 metabolism of linoleic acid, inhibits cell proliferation and induces cell cycle arrest and apoptosis in transformed colonic epithelial cells [105]. 15-LOX-1 protein expression and 13-S-HODE intracellular levels are decreased in human colonic tumors [105]. Shureiqi et al. showed that 13-S-HODE bound to PPAR*δ* and then downregulated PPAR*δ* expression and activation in colorectal cancer cells, DLD-1 and RKO, and that the loss of PPAR*δ* expression in HCT116 markedly suppressed 13-S-HODE-mediated apoptosis [106]. 15-LOX-1 expression also downregulated PPAR*δ* expression and transcriptional activity in these colorectal cancer cells [106]. Furthermore, NSAIDs increase 15-LOX-1 protein expression and its product 13-S-HODE levels and downregulate PPAR*δ* expression in association with subsequent growth inhibition and apoptosis [106, 107]. Thus it is considered possible that PPAR*δ* promotes the growth of colon cancers.

On the contrary, other reports suggest that ligand activation of PPAR*δ* promotes the induction of terminal differentiation and inhibition of cell growth. PPAR*δ* was found in intestinal epithelial cells in both the normal intestine and adenomas of *A* *p* *c* ^min^ mice [101]. Reed et al. reported that targeted deletion of the APC alleles in mouse intestines decreased the expression levels of PPAR*δ* mRNA and protein, although *β*-catenin and c-*myc* were increased [108]. Marin et al. showed that PPAR*δ* expression was reduced in both the *A* *p* *c* ^min^ mouse colon polyps and azoxymethane (AOM)-treated wild-type mouse polyps, though the expression levels of PPAR*δ* mRNA in colonic epithelium were not different between *A* *p* *c* ^min^ mice and wild-type mice with or without AOM-treatment [109]. Several reports identified that the transcription factor binding sites for AP-1, CCAAT/enhancer-binding proteins, vitamin D receptor, and others were found in human or mouse PPAR*δ* promoter, and these transcription factors regulated PPAR*δ* expression [16, 110, 111]. However, further investigation is required to certify the regulation of PPAR*δ* expression in cancer.

Hollingshead et al. reported that GW501516 and GW0742, highly specific PPAR*δ* ligands, did not increase the growth of human colon cancer cell lines (HT-29, HCT116, and LS-174T) and liver cancer cell lines (HepG2 and HuH7) cultured in the presence or absence of serum [112]. In addition, treatment of these cell lines with either GW501516 or GW0742 did not change the phosphorylation of Akt, and no increase in the expression levels of COX2 or VEGF were detected [112]. Similar results were observed in the colon or liver of *A* *p* *c* ^min^ mice treated with GW501516 or GW0742 [109, 112]. Barak et al. showed that the average number of intestinal polyps was not significantly different between *PPARd* ^+/+^/*A* *p* *c* ^min^, *PPARd* ^+/−^/*A* *p* *c* ^min^, and *PPARd* ^−/−^/*A* *p* *c* ^min^ mice, although this study was limited to a small number [113]. On the other hand, several studies showed that colon polyp formation was enhanced in the absence of PPAR*δ* expression in both *PPARd* ^−/−^/*A* *p* *c* ^min^ and AOM-treated *PPARd* ^−/−^ mice [108, 109, 114]. Moreover, Marin et al. showed that the administration of GW0742 had no effect on colon or small intestinal tumorigenesis in either *PPARd* ^−/−^/*A* *p* *c* ^min^ or *PPARd* ^+/+^/*A* *p* *c* ^min^ mice as compared with controls [109]. In addition, decrease of colon polyp multiplicity was observed in *PPARd* ^+/+^ AOM-treated mice administrated with GW0742 compared with control wild-type mice. This effect was likely due in part to PPAR*δ*-dependent induction of colonocyte differentiation and enhancement of apoptosis [109]. Indeed, PPAR*δ* induces keratinocyte terminal differentiation, which normally opposes cell proliferation [115, 116]. Hatae et al. also showed that intracellular PGI_2_, an endogenous PPAR*δ* ligand, formed by expressing PGIS in human embryonic kidney 293 (HEK293) cells, promoted apoptosis by activating PPAR*δ* [117]. In this way, PPAR*δ* inhibits tumor growth by inducing apoptosis or differentiation.

Thus the conflicting reports in the literature suggest that PPAR*δ* either potentiates or attenuates colon cancer. Similar discrepancies were observed in other tissues. Di-Poï et al. showed that the activation of PPAR*δ* inhibited apoptosis in keratinocyte [118]. The activation of PPAR*δ* by L-165041, one type of PPAR*δ* ligand, upregulates 3-phosphoinositide-dependent kinase-1 (PDK1) and integrin-linked kinase (ILK) gene expression via PPRE and downregulates phosphatase and tensin homolog (PTEN) protein expression, and subsequently leads to the activation of Akt1 in a PI3K- dependent manner in mouse primary keratinocytes and human keratinocyte HaCaT cells [118]. Yin et al. showed that PPAR*δ* ligand GW501516 accelerated progestin- and carcinogen-induced mouse mammary carcinogenesis [119]. Stephen et al. reported that PPAR*δ* selective agonists stimulated the proliferation of human breast and prostate cancer cell lines and primary endothelial cells [104]. On the other hand, Burdick et al. reported that ligand activation of PPAR*δ* with GW0742 inhibited the cell growth of either human keratinocyte cell line N/TERT1 or mouse primary keratinocytes [120]. In these cells, ligand activation of PPAR*δ* by GW0742 did not alter expression and/or modulation of the PTEN/PDK1/ILK1/Akt pathway [120]. Girroir et al. reported that both GW0742 and GW501516 inhibited the growth of the human breast cancer cell line, MCF7, and human melanoma cell line, UACC903 [121].

**Figure 2:**
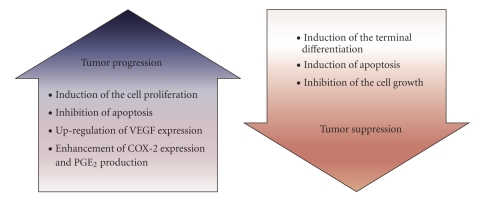
Does PPAR*δ* progress or suppress tumor growth?

To date, however, the reason for the contradiction in these observations is unclear. One explanation for these conflicting results may be the ability of PPAR*δ* to repress the transcription of target genes. We and others observed that unliganded PPAR*δ* repressed target gene expression, though ligand-activated PPAR*δ* induced these genes [30, 122–124]. It has been reported that unliganded PPAR*δ* bound to PPRE and recruited corepressors, such as B-cell lymphoma 6 (BCL-6), silencing mediator for retinoid and thyroid hormone receptor (SMRT), nuclear receptor corepressor (NCoR), and others. On the other hand, liganded PPAR*δ* is thought to release the corepressor and form a complex with coactivators [122–124]. Furthermore, binding of ligand to the PPAR*δ* or deletion of PPAR*δ* expression may lead to the release of BCL-6. Subsequently, BCL-6 represses the transcription of a number of inflammatory cytokine genes [124]. Thus the PPAR*δ* activity may be influenced by the cellular environment, such as the existence of PPAR*δ* ligands, cofactors, and others. From this viewpoint, the conflicting results may be due to differences in the condition of cell cultures or the genetic background of animal models. Secondly, prostaglandins, some of which act as PPAR ligands, have a variety of biological activities. Prostaglandins, synthesized via the COX pathway from arachidonic acid, are released outside the cells and lead to changes in the cellular levels of cyclic AMP and Ca^2+^ through binding to G-protein-coupled receptors on the plasma membrane [90]. Indeed, Hatae et al. suggested that cAMP produced by the PGI_2_-PGI receptor (IP)-cAMP pathway might protect vascular endothelial cells from intracellular PGI_2_-PPAR*δ*-mediated apoptosis [117]. On the other hand, Fauti et al. showed that the ectopic expression of COX-2 and PGIS in HEK293 cells results in a dramatic induction of PGI_2_ synthesis, but no increase in PPAR*δ* transcriptional activity is observed [125]. Thus they suggest that PGI_2_ lacks agonistic activity for PPAR*δ*. Since PGI_2_ is unstable and rapidly hydrolyzed to 6-keto-PGF_1*α*_ within minutes and increases the production of intracellular cAMP via stimulation of adenylyl cyclase through the cell surface IP receptor, further investigation is necessary to certify the mechanism of the effect of the PGI_2_ on PPAR*δ* activity in detail. Therefore, additional analyses are necessary to define the PPAR*δ* functions in cancer (Figure 2).

## 4. PPAR*γ* AND CANCER

Cancer cells represent dysregulaton of the cell cycle and lead to cell proliferation. In this viewpoint, modulators of the cell cycle and/or apoptosis are useful as chemotherapeutic agents for cancer [126, 127]. A number of investigators have shown that PPAR*γ* was expressed in a variety of tumor cells, and the activation of PPAR*γ* by ligands led to either inhibition of cell proliferation or induction of apoptosis (Table 2) [128, 129]. PPAR*γ* is expressed in colonic tumors, normal colonic mucosa, and colon cancer cell lines [130–135]. Kitamura et al. showed that TZDs, such as troglitazone and rosiglitazone, inhibited the cell growth and induced G1 cell cycle arrest of rat intestinal epithelial cells [136]. The cell growth inhibition by TZDs was caused by the decrease of the expression of cyclin D1, critical for entering the S phase of the cell cycle. TZDs suppressed the cyclin D1 promoter activity through inhibition of the transcriptional activities of AP-1 and Ets [136]. Shao et al. demonstrated that treatment with rosiglitazone inhibited the K-Ras-induced elevation of the expression levels of cyclin D1 by inhibition of the K-Ras-induced phosphorylation of Akt, resulting in the G1 cell cycle arrest [89]. Furthermore, J.-A. Kim et al. showed that treatment of the human colorectal cell line, HCT15, with troglitazone induced the expression of p21^Cip1/Waf1^, that is, a CDK inhibitor (CKI) and negatively regulates the cell cycle progression, through the ERK pathway, and inhibited HCT15 cell growth [155]. PPAR*γ* ligands also induce apoptosis in human colon cancer cells [156]. Chen et al. showed that PPAR*γ* ligands, 15-Deoxy-Δ^12,14^-prostaglandin J_2_⁢ (15dPGJ_2_), or ciglitazone, induced apoptosis in HT-29 by inhibiting nuclear factor kappa B (NF-*κ*B) activity, which upregulates various antiapoptotic genes, and suppressing the expression of BCL-2, which protects cells against apoptosis [133]. Furthermore, using the in vivo mouse model, the administration of TZD to mice reduced AOM and/or dextran sodium sulfate-induced formation of aberrant crypts foci and colon carcinogenesis [131, 157]. In addition, PPAR*γ* ligands also inhibit the cell growth of several breast cancer cell lines and mammary gland tumor development [137, 158–162]. Elstner et al. showed that PPAR*γ* ligands, troglitazone, 15dPGJ_2_, and indomethacin, caused inhibition of proliferation in several human breast cancer cell lines, such as MCF7, MDA-MB-231, BT474, and T47D [162]. Troglitazone also inhibited MCF7 tumor growth in triple-immunodeficient BNX nude mice [162]. Clay et al. reported that 15dPGJ_2_ and troglitzaone attenuated cellular proliferation of MDA-MB-231 by blocking cell cycle progression and inducing apoptosis [160]. Pretreatment of MDA-MB-231 cells with 15dPGJ_2_ attenuated the capacity of these cells to induce tumors in nude mice [160]. Yin et al. showed that treatment of MCF7 with troglitazone also decreased the expression of several regulators of pRb phosphorylation, such as cyclin D1, CDK4, CDK6, and CDK2 [158]. pRB is a retinoblastoma tumor suppressor gene product, and phosphorylated pRB induces cell cycle progression [163]. Troglitazone induced the G1 cell cycle arrest by attenuation of pRb phosphorylation, resulting in inhibition of cell proliferation [158]. Suh et al. showed that GW7845, synthetic PPAR*γ* ligand, prevented mammary carcinogenesis in the rat model that used nitrosomethylurea as the carcinogen [159]. Mehta et al. also reported that troglitazone prevented the induction of preneoplastic lesions by 7, 12-dimethylbenz[*a*]anthracene in a mouse mammary gland organ culture model [161]. Moreover, PPAR*γ* ligands inhibit the cell proliferation in other types of cancer. PPAR*γ* ligands inhibited the growth of esophageal squamous carcinoma cell lines by inducing G1 arrest associated with an increased level of several CKIs, such as p27^Kip1^, p21^Cip1/Waf1^, and p18^Ink4c^ [138]. PPAR*γ* ligands also induced apoptosis and G1 cell cycle arrest in human gastric cancer cells, and that inhibited cell proliferation [139, 164]. In human pancreatic cancer cells, PPAR*γ* ligands induced apoptosis and growth inhibition associated with G1 cell cycle arrest through increasing p27^Kip1^ protein expression [140, 165–167]. In human hepatocellular carcinoma cell lines, PPAR*γ* ligands induced cell cycle arrest through increased expression of p21^Cip1/Waf1^, p27^Kip1^, and p18^Ink4c^ protein levels [141, 168]. Troglitazone also induced the activation of the cell death protease, caspase 3, and that induced apoptosis of human liver cancer cell lines [169]. PPAR*γ* is abundantly expressed in human adrenal tumors including adrenocortical carcinomas and normal adrenal tissues. PPAR*γ* agonists suppress adrenocortical tumor cell proliferation, increase apoptosis, and induce adrenal differentiation [142, 170]. Moreover, PPAR*γ* ligand showed antitumor effect against human prostate cancer cells and human lung cancer cells [143, 144, 171–173]. Thus PPAR*γ* ligands could suppress the tumorigenesis. Therefore, PPAR*γ* ligands could be used as antineoplastic drugs.

In contrast, both troglitazone and rosiglitazone treatment increased the frequency and size of colon tumors in *A* *p* *c* ^min^ mice [174, 175]. Treatment with rosiglitazone also increased the expression levels of *β*-catenin, a protein involved in Wnt signaling and correlating with enhanced cell proliferation, in the colon of *A* *p* *c* ^min^ mice and HT-29 cells [174]. To investigate the basis for this contradiction, Girnun et al. used mice heterozygous for PPAR*γ* with both chemical and genetic models of human colon cancer [176]. Heterozygous loss of PPAR*γ* caused a greater incidence of colon cancer when these mice were treated with AOM. Although there was no difference in *β*-catenin expression levels in colorectal tumors between AOM-treated *PPARg* ^+/−^ and wild-type mice, *β*-catenin expression levels in the colonic epithelium of untreated *PPARg* ^+/−^ mice were greater than that of untreated wild-type mice. When crossing to *A* *p* *c* ^1638*N*^ mice, the mouse model for FAP, there were also no difference in *β*-catenin levels between *PPARg* ^+/−^/*A* *p* *c* ^1638*N*^ and *PPARg* ^+/+^/*A* *p* *c* ^1638*N*^ mice before polyp formation. Survival and the number of tumors formed in the colon also showed no difference in both mice. Thus although PPAR*γ* has the potential to suppress *β*-catenin levels and colon carcinogenesis, PPAR*γ* has no effect on *β*-catenin levels or tumorigenesis in the presence of APC signaling dysfunction [176]. Furthermore, PPAR*γ* mutations, some of which show the loss of the transactivation ability, are found in colon cancers in humans, and that PPAR*γ* may be considered as a tumor suppressor gene [134]. On the other hand, to evaluate the contribution of PPAR*γ* to breast cancer, Saez et al. generated transgenic mice, MMTV-VpPPAR*γ* mice, that express a constitutively active form of PPAR*γ* in mammary gland [177]. MMTV-VpPPAR*γ* mice showed normal development of mammary gland and no increased tendency to develop tumors. To assess the influence of increased PPAR*γ* signaling on mammary gland neoplasia, MMTV-VpPPAR*γ* mice were crossed to mice that express a polyoma virus middle T antigen (PyV-MT) in mammary tissue, MMTV-PyV mice, which rapidly develop tumors. These mice that expressed both activated PPAR*γ* and PyV-MT showed accelerated development of mammary tumors. Therefore, although increased PPAR*γ* activation does not initiate tumor formation in normal mammary tissue, once a tumor-initiating event occurs, PPAR*γ* signaling serves as a tumor promoter in the mammary gland. Furthermore, there is no difference in tumor development between MMTV-PyV mice and the mice, generated by crossing *PPARg* ^+/−^ mice to MMTV-PyV mice [177]. Thus in this model, PPAR*γ* does not act as a tumor suppressor gene.

Furthermore, PPAR*γ* ligands exert their biological effects through a PPAR*γ*-independent pathway. Palakurthi et al. reported that troglitazone and ciglitazone induced G1 arrest by inhibiting translation initiation in both *PPARg* ^−/−^ and *PPARg* ^+/+^ mouse embryonic stem cells. Thus TZDs inhibit cell proliferation and tumor growth in a PPAR*γ*-independent manner [178]. Therefore, although PPAR*γ* ligands are used as insulin sensitizers, further investigation is needed to clarify whether PPAR*γ* ligands are effective chemotherapeutic agents for cancer in humans.

## 5. SUMMARY

PPARs are linked to metabolic disorders and are interesting pharmaceutical targets. Among the synthetic ligands, fibrates are hypolipidemic compounds that activate PPAR*α*, and TZDs, which selectively activate PPAR*γ*, are hypoglycaemic molecules that are used to treat type 2 diabetes. PPAR*δ* agonists might form effective drugs for obesity, diabetes, and cardiovascular disease. Moreover, recent evidence suggests that PPAR modulators may have beneficial effects as chemopreventive agents [179]. However, as mentioned above, it remains unclear whether PPARs act as oncogenes or as tumor suppressors. From this viewpoint, current strategies are aimed at reducing the side effects and improving the efficacy and safety profile of PPAR agonists, termed selective PPAR modulators (SPPARMs) [180, 181]. This model proposes that SPPARMs bind in distinct manners to the ligand binding pocket of PPAR and induce distinct conformational changes of the receptor, resulting in differential interactions with cofactors according to the combination of their expression levels in different organs. Thus each SPPARM leads to differential gene expression and biological response. However, what kinds of cofactors are recruited to PPAR by each SPPARM is still unknown. Thus it is important to identify the cofactor complex for PPAR with each SPPARM and the expression patterns of cofactors in various tissues. Furthermore, recent evidence suggests that the ligand binding protein in the cytosol that transports ligands into the nucleus is important to modulate the action of nuclear receptors. Long-chain fatty acids, endogenous PPAR ligands, are highly hydrophobic and fatty acids are bound to fatty acid binding proteins (FABPs) in the aqueous intracellular compartment [182]. FABPs also bind to PPAR ligands and transport them from the cytosol into the nucleus [183–191]. In the nucleus, FABPs interact directly with PPARs and deliver ligands to PPARs, and the activity of PPARs is modulated [186, 187, 190–192]. Recently, Schug et al. showed that when the cellular retinoic acid binding protein-II (CRABP-II) expression levels were higher than FABP5 in the cells, retinoic acid (RA) bound to CRABP-II. Subsequently, CRABP-II relocated to the nucleus and delivered RA to RAR, resulting in inhibition of cell proliferation and induction of apoptosis [187, 193]. On the contrary, when the FABP5 to CRABP-II ratio is high, RA serves as a physiological ligand for PPAR*δ*, which induces cell survival and proliferation [187, 194]. Therefore, it is important to identify the cytosolic ligand binding proteins and the expression levels of the proteins for defining the physiological effects of ligands. Furthermore, several ligands exert their biological effects through a PPAR-independent pathway [195]. Thus further studies are required to elucidate the role of PPARs for developing new efficiently and safety chemotherapeutic agents for cancer.

## Figures and Tables

**Table 1:** Summary of the species differences of PPAR*α*.

	Human	Rodent
PPAR*α* expression levels	+	++
PPAR*α* variants	Yes	?
Peroxisome proliferation	+/−	+
Fatty acid metabolism	+	+
Expression of cell cycle regulator genes	+/−	+
Expression of miRNA (let-7C)	+	−
Hepatocellular proliferation	+/−	+
Apoptosis	+	−
Liver tumor	+/−	++

**Table 2:** The expression of PPAR*γ* in cancer.

	References
Colonic tumor	[135]
Breast tumor	[137]
Esophageal tumor	[138]
Gastric cancer	[139]
Pancreatic cancer	[140]
Hepatocellular carcinoma	[141]
Adrenocortical carcinoma	[142]
Lung tumor	[143]
Prostate cancer	[144]
Liposarcoma	[145]
Thyroid carcinoma	[146]
Bladder cancer	[147]
Renal cell carcinoma	[148]
Melanoma	[149]
Squamous cell carcinoma	[150]
Cervical carcinoma	[151]
Testicular cancer	[152]
Neuroblastoma	[153]
Pituitary tumor	[154]
